# Evaluation of Patterns and Correlations of the Degree of Conjunctival Hyperemia Induced by Omidenepag Isopropyl 0.002% and Ripasudil 0.4%

**DOI:** 10.7759/cureus.10368

**Published:** 2020-09-10

**Authors:** Etsuko Terao, Shunsuke Nakakura, Yuki Nagata, Saki Dote, Hitoshi Tabuchi, Yoshiaki Kiuchi

**Affiliations:** 1 Ophthalmology, Saneikai Tsukazaki Hospital, Himeji, JPN; 2 Ophthalmology, Hiroshima University, Hiroshima, JPN

**Keywords:** omidenepag isopropyl ophthalmic solution 0.002%, ripasudil 0.4%, glaucoma, conjunctival hyperemia

## Abstract

Purpose: To evaluate the pattern of conjunctival hyperemia induced by omidenepag isopropyl 0.002% and ripasudil 0.4%, and its correlation with the degree of hyperemia.

Subjects and Methods: We previously reported the time course of conjunctival hyperemia induced by administering one drop of omidenepag isopropyl to one eye and one drop of ripasudil to the other eye in 34 healthy subjects (mean age: 29.7 years; 22 females, 12 males). We assessed the degree of hyperemia by slit-lamp photography of the frontal and temporal conjunctiva 0, 15, 30, 60, 120, 180, and 360 min after the administration of one drop of omidenepag isopropyl and ripasudil. The data were used to compare the frontal photographs before and at the peak of hyperemia according to the clinical hyperemia score (0-3) and classify the pattern of developing hyperemia due to both drugs. We also examined the correlation between the degree of hyperemia by comparing the images captured at the peak of hyperemia in both groups, using clinical hyperemia score and “percent coverage” of conjunctival hyperemia by using an automated hyperemia analysis software program; this program provides the pixel coverage of the conjunctival vessels in the region of interest.

Results: There were significant differences in the developmental pattern of hyperemia between omidenepag isopropyl-administered and ripasudil-administered eyes (P<0.001, χ^2^ test), with dilation of large blood vessels only (N=2 vs. 1, respectively), small blood vessels only (N=17 vs. 5), both large and small blood vessels (N=8 vs. 27), and no change (N=6 vs. 0). The degree of hyperemia between the two groups was positively correlated with the hyperemia score (rs=0.344, P=0.055) in the frontal conjunctival photographs and the percent coverage of conjunctival blood vessels (r=0.510, P=0.003) in the temporal conjunctival photographs.

Conclusions: The pattern of conjunctival hyperemia induced by omidenepag isopropyl predominantly involved small blood vessels, whereas that of ripasudil involved both large and small blood vessels. The eyes that were hyperemic with omidenepag isopropyl also tended to be hyperemic with ripasudil.

## Introduction

Glaucoma is a major cause of blindness worldwide, currently affecting approximately 60 million individuals [1]. Currently, there is a wide variety of glaucoma ophthalmic solutions available. In 2018, the selective prostaglandin EP2 receptor agonist omidenepag isopropyl 0.002% (EYBELIS®; Santen Pharmaceutical, Co., Ltd., Osaka, Japan) was introduced in Japan. Its intraocular hypotensive effect has been demonstrated in humans and is not inferior to that of latanoprost 0.005% [2-4]. Unlike the side effects of conventional prostaglandin F2α products, those of omidenepag isopropyl are unique and have been reported up to six months after market launch. Side effects, in 580 patients with 809 cases, included conjunctival hyperemia (N=187), iritis (N=86), blurred vision (N=81), eye pain (N=43), visual impairment (N=48), macular edema (N=23), and myopia (N=16) [5]. Among these, conjunctival hyperemia was the most common side effect of omidenepag isopropyl.

A previous report showed that, among the side effects of conventional eye drops, conjunctival hyperemia (35.6%) was the most common complaint of patients [6]. Therefore, conjunctival hyperemia is considered important for patient adherence to the treatment of glaucoma. On the other hand, conjunctival hyperemia was the most frequent side effect (>60%) in patients administered with the relatively newer Rho-kinase inhibitor ripasudil 0.4% (Glanatec®; Kowa Co., Ltd., Aichi, Japan) [7-10]. Thus, it is noteworthy that the most common side effect of these two newer anti-glaucoma eye drops is conjunctival hyperemia. Recently, we reported the difference in the time course of conjunctival hyperemia in healthy subjects who received one drop of omidenepag isopropyl and ripasudil. Using slit-lamp photography, we showed that hyperemia was less severe and resolved more rapidly with omidenepag isopropyl than ripasudil [11]. The purpose of this study was to determine the patterns of conjunctival hyperemia and the correlation between the degree of hyperemia caused by omidenepag isopropyl and ripasudil.

## Materials and methods

**Figure 1:**
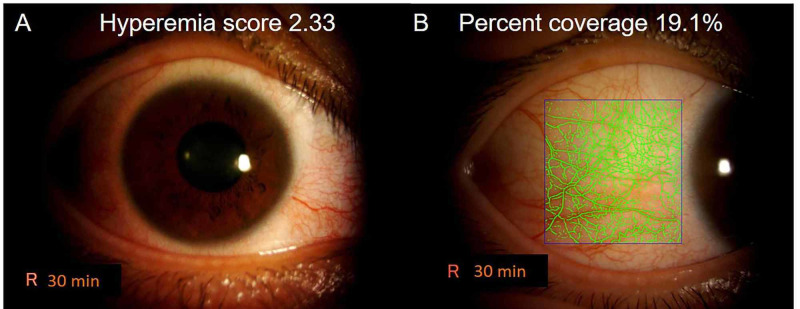
Conjunctival hyperemia evaluation method. A: Conjunctival hyperemia score of 2.33 on a frontal image at 30 min in a 36-year-old male induced by omidenepag isopropyl. B: Conjunctival vessel occupancy (percent coverage). Percent coverage was 19.1% (according to the analysis software) at the same time point.

This study was approved by the ethics committee of Sanei-kai Tukazaki Hospital (Himeji, Japan) and conducted in accordance with the tenets of the Declaration of Helsinki [12]. The subjects were 34 healthy individuals (mean age: 29.7 years; 22 females, 12 males) from a previous study [11]. Frontal and temporal conjunctiva were photographed 0, 15, 30, 60, 120, 180, and 360 min after the administration of one drop of omidenepag isopropyl in the right eye and ripasudil in the left eye [11]. Slit-lamp images were captured using an SL-D7 camera (TOPCON, Tokyo, Japan). The detailed slit-lamp photographic conditions were as follows. The angle between the slit lamp and the microscope arm was set at 30°. The camera flash light was adjusted to level one. The slit width was set at 20 mm, and the objective magnification was set at 10×. The diffuser of the slit lamp was used. In the previous study, the degree of hyperemia was analyzed in two different ways. The frontal images were used for subjective analysis and were categorized according to the Japanese Allergic Conjunctivitis guidelines into four levels: 0, none; 1, mild; 2, moderate; and 3, severe [13]. Three raters performed independent assessments, and the average of their scores was used to determine the hyperemia score (Figure 1A). Additionally, a hyperemia analysis software was used to determine the degree of hyperemia of the temporal conjunctiva [14,15]. This software measures the occupancy of the vessels in the region of interest as percent coverage (%) of conjunctival blood vessels (Figure 1B); the reproducibility of this software was high (R2=0.79) [14,15]. The temporal conjunctiva (700-890 pixels [width] × 800-920 pixels [height]) was assessed for objective analysis in all subjects because it is the widest area among the four conjunctival fields (superior, inferior, nasal, and temporal). The measured location and area were constant in each individual [11].　

Pattern classification of conjunctival hyperemia

**Figure 2:**
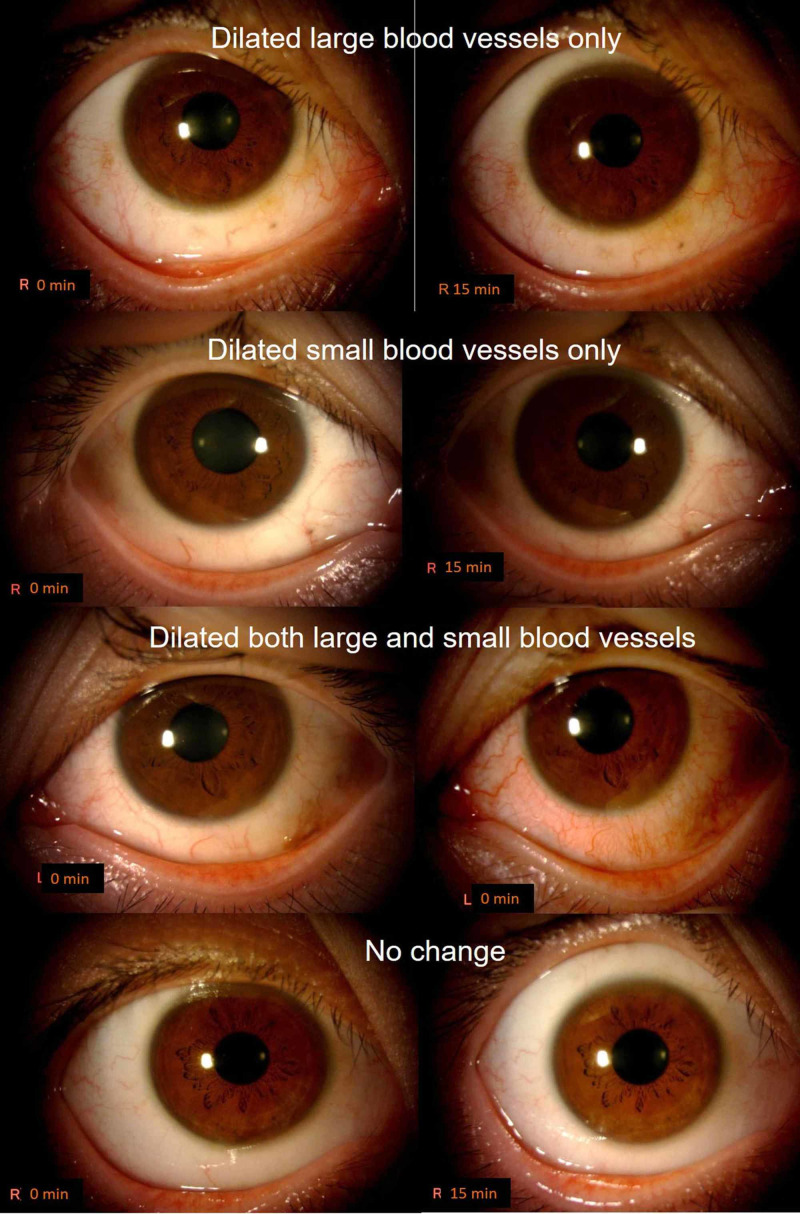
Classification of the conjunctival hyperemia pattern. Dilated large vessels only, dilated small vessels only, dilated both large and small vessels, and no change.

Frontal images were used for the classification of hyperemia. Firstly, two eyes of different patients (one eye per treatment) that did not exhibit any changes over time following treatment with omidenepag isopropyl and ripasudil were excluded based on the seven consecutive anterior images obtained from 0 to 360 min. In the remaining 66 eyes, the changes in hyperemia were judged by comparing the slit-lamp photographs captured prior to the initiation of the eye drop administration and at the time of the peak hyperemia score. If the hyperemia score was constant (e.g., same score at 15, 30, and 60 min), the image of the first peak time (15 min) was used. To the best of our knowledge, there is no previous report on the pattern of hyperemia. Therefore, the first author (E.T.) compared all the images and classified them into four patterns: dilated large vessels only, dilated small vessels only, dilated both large and small vessels, and no change (Figure 2). Subsequently, each independent evaluator (E.T., Y. N., and S.D.) performed this categorization using a scoring sheet by comparing the baseline images with those captured at the peak time. If the categorization was consistent between at least two of the three evaluators, it was used as the final categorization. In short, two evaluators categorized as (dilated large vessels only) and one scored (dilated small vessels only), the result was judged as “dilated large vessels only”. If the decision was separated by three evaluators each, they judged the photographs again.

Evaluation of the correlation of conjunctival hyperemia induced by omidenepag isopropyl and ripasudil

Two patients whose hyperemia scores did not change in the frontal photographs were excluded. Each of the 32 remaining patients was correlated with the peak hyperemia scores in omidenepag isopropyl-administered and ripasudil-administered eyes. For the evaluation of the percent coverage of the temporal conjunctiva, four eyes that did not demonstrate an increase in percent coverage after administered with omidenepag isopropyl were excluded. Therefore, by excluding the contralateral eye of the same four patients, the conjunctival hyperemia correlation between the eyes was eventually evaluated in 30 patients.

Statistical analysis

The χ2 test was used to determine differences in the frequency of omidenepag isopropyl and ripasudil eyes in the classification of hyperemia patterns. The Spearman’s rank correlation coefficient test was used to determine the degree of conjunctival hyperemia with respect to the hyperemia score (ordinal scale). Pearson’s correlation coefficient was used to determine the correlation for the percent coverage of temporal conjunctiva because it represents a normal distribution in healthy subjects [14,15]. P-values <0.05 denoted statistically significant differences. A correlation coefficient of r/rs between 0.2 and 0.4 denotes a weak positive correlation, 0.4-0.7 denotes moderate positive correlation, and 0.7-1.0 denotes a strong positive correlation.

## Results

Classification of the conjunctival hyperemia pattern

Nine eyes administered with omidenepag isopropyl and two eyes administered with ripasudil were re-evaluated, and all eyes had final decision at the second time judgments. The results showed that, among the omidenepag isopropyl eyes, there were two eyes with large blood vessels only, 17 eyes with small blood vessels only, eight eyes with both large and small blood vessels, and six eyes with no change. Among the ripasudil-administered eyes, one eye had large vessels only, five eyes had small vessels only, 27 eyes had both large and small blood vessels, and 0 eyes remained unchanged (Table 1). A significant difference in distribution was found between the two groups according to the χ2 test (P<0.001). In omidenepag isopropyl-administered eyes, hyperemia with small vessels only (N=17) was the most frequently observed pattern (51%). In ripasudil-administered eyes, hyperemia with both large and small vessels (N=27) was the most frequently noted pattern (81%). Thus, the difference in hyperemia reported in ripasudil-administered eyes versus omidenepag isopropyl-administered eyes was related to the greater number of dilated large vessels in the conjunctiva.

**Table 1:** Differences in the distribution of hyperemia patterns in omidenepag isopropyl- and ripasudil-administered eyes A significant difference in distribution was found between the two groups according to the χ2 test (P<0.001).

	Omidenepag isopropyl, N=33 eyes	Ripasudil, N=33 eyes
Dilated large blood vessels only	2	1
Dilated small blood vessels only	17	5
Dilated both large and small blood vessels	8	27
No change	6	0

Evaluation of the correlation between the degree of conjunctival hyperemia in eyes administered with omidenepag isopropyl and ripasudil

**Figure 3:**
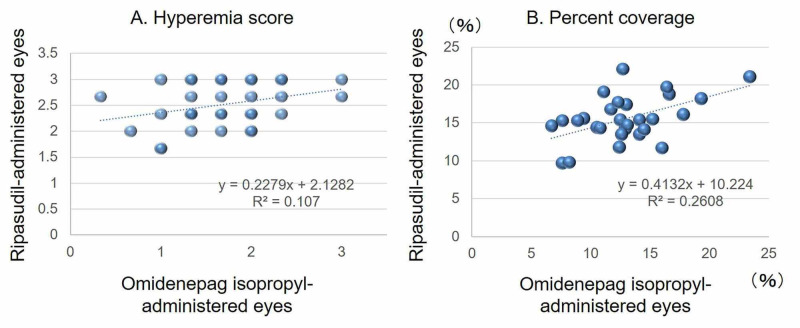
Correlation between the degree of conjunctival hyperemia in eyes administered with omidenepag isopropyl and ripasudil. A: Conjunctival hyperemia score revealing a weak positive correlation (rs=0.344, P=0.055) (N=32). B: Percent coverage revealing a moderately positive correlation (r=0.510, P=0.003) (N=30).

In the analysis of correlations using the conjunctival hyperemia score, a weak positive correlation (rs=0.344) was found; however, the statistical reliability of rs was not significant (P=0.055, Spearman’s rank correlation coefficient test) (Figure 3A). In the analysis of correlations using the percent coverage calculated by hyperemia analysis software showed a moderately positive correlation (r=0.510), and the reliability of the correlation coefficient was statistically significant (P=0.003, Pearson’s correlation coefficient test) (Figure 3B). In other words, eyes that were hyperemic with omidenepag isopropyl were also likely to be hyperemic with ripasudil.

## Discussion

In the present study, we evaluated different patterns of conjunctival hyperemia induced by omidenepag isopropyl and ripasudil. We found that hyperemia was more severe in ripasudil-administered eyes than in omidenepag isopropyl-administered eyes due to the dilation of large blood vessels in the conjunctiva. Additionally, the degree of conjunctival hyperemia in omidenepag isopropyl-administered eyes positively correlated with that observed in ripasudil-administered administered eyes. This suggests that patients with hyperemia after treatment with omidenepag isopropyl may also be hyperemic after treatment with ripasudil. Our previous report showed that the peak hyperemia score (mean) was 1.57 at 30 min and 2.42 at 15 min after administration of omidenepag and ripasudil, respectively [11]. Similarly, the peak of hyperemia in percent coverage (mean) was 11.91% at 30 min and 15.26% at 15 min, respectively. The tendency for conjunctival hyperemia after treatment with omidenepag isopropyl was slower and weaker than that noted after the administration of ripasudil. We hypothesized that this difference in hyperemia score and percent coverage is due to the dilation of large vessels by ripasudil. The exact cause of omidenepag isopropyl-induced hyperemia is currently unknown. However, it has been reported in rabbit eyes that stimulation of EP2 receptors relaxes smooth muscle and increases blood flow to the retina and choroid, causing retinal vascular dilation [16]. This effect may also occur in the conjunctival vessels. On the other hand, ripasudil has been shown to relax smooth muscle in conjunctival vessels and modulate vascular endothelial cells [17-19]. It is speculated that smooth muscle relaxation is an effect of both drugs; however, the reasons responsible for the differences between these drugs remain unclear. To the best of our knowledge, there has been no previous study examining the distribution of conjunctival blood vessels according to age, sex, race, or left and right sides. If it assumes that there is no difference in the distribution of small and large conjunctival blood vessels between the left and right eyes in healthy individuals, the results of this study emphasize the fact that patients who experience hyperemia after treatment with omidenepag isopropyl may also be hyperemic following the administration of ripasudil. A limitation of this study is that the agreement of hyperemia scores determined by humans is inevitably low (intraclass correlation value between the three raters was as low as 0.391 in our data) [11], unlike the evaluations performed using hyperemia analysis software. Therefore, the correlation coefficient may be reduced when assessing the degree of hyperemia score (rs=0.344, P=0.055). The second, categorization of the blood vessels as large or small, is mainly subjective because all the conjunctival vessels are connected and cannot be divided by direct measurements of vessel diameter. Additionally, some blood vessels may be scleral vessels. 

## Conclusions

There was a difference in the pattern of conjunctival hyperemia induced by omidenepag isopropyl and ripasudil. Conjunctival hyperemia with omidenepag isopropyl was associated with small vessels, whereas the conjunctival hyperemia with ripasudil was linked to both large and small vessels. The eyes that were hyperemic with omidenepag isopropyl also tended to be hyperemic with ripasudil. Conjunctival hyperemia is the most common complaint of patients. Therefore, our results will be useful for ophthalmologists to manage patients' adherence with glaucoma. 

## References

[1] Quigley HA (2011). Glaucoma. Lancet.

[2] Duggan S (2018). Omidenepag isopropyl ophthalmic solution 0.002%: first global approval. Drugs.

[3] Aihara M, Lu F, Kawata H (2019). Phase 2, randomized, dose-finding studies of omidenepag isopropyl, a selective ep2 agonist, in patients with primary open-angle glaucoma or ocular hypertension. J Glaucoma.

[4] Lu FH, Aihara M, Kawata H (2018). A phase 3 trial comparing omidenepag isopropyl 0.002% with latanoprost 0.005% in primary open-angle glaucoma and ocular hypertension: the AYAME study. Invest Ophthalmol Vis Sci.

[5] (2020). Santen. https://www.santen.co.jp/medical-channel/di/safety/DI050_safety_eybelis.pdf.

[6] Park MH, Kang KD, Moon J (2013). Korean Glaucoma Compliance Study Group: noncompliance with glaucoma medication in Korean patients: a multicenter qualitative study. Jpn J Ophthalmol.

[7] Tanihara H, Inoue T, Yamamoto T, K-115 Clinical Study Group (2014). Phase 1 clinical trials of a selective Rho kinase inhibitor, K-115. JAMA Ophthalmol.

[8] Tanihara H, Inoue T, Yamamoto T, K-115 Clinical Study Group (2013). Phase 2 randomized clinical study of a Rho kinase inhibitor, K-115, in primary open-angle glaucoma and ocular hypertension. Am J Ophthalmol.

[9] Tanihara H, Inoue T, Yamamoto T, K-115 Clinical Study Group (2015). Additive intraocular pressure-lowering effects of the Rho kinase inhibitor ripasudil (K-115) combined with timolol or latanoprost: a report of 2 randomized clinical trials. JAMA Ophthalmol.

[10] Tanihara H, Inoue T, Yamamoto T, K-115 Clinical Study Group (2015). Intra-ocular pressure-lowering effects of a Rho kinase inhibitor, ripasudil (K-115), over 24 hours in primary open-angle glaucoma and ocular hypertension: a randomized, open-label, crossover study. Acta Ophthalmol.

[11] Terao E, Nakakura S, Fujisawa Y (2020). Time course of conjunctival hyperemia induced by omidenepag isopropyl ophthalmic solution 0.002%: a pilot, comparative study versus ripasudil 0.4%. BMJ Open Ophthalmol.

[12] (2020). Declaration of Helsinki. https://www.wma.net/what-we-do/medical-ethics/declaration-of-helsinki/..

[13] Takamura E, Uchio E, Ebihara N (2017). Japanese guidelines for allergic conjunctival diseases. Japanese Society of Allergology. Allergol Int.

[14] Yoneda T, Sumi T, Hoshikawa Y (2019). Hyperemia analysis software for assessment of conjunctival hyperemia severity. Curr Eye Res.

[15] Yoneda T, Sumi T, Takahashi A (2012). Automated hyperemia analysis software: reliability and reproducibility in healthy subjects. Jpn J Ophthalmol.

[16] Mori A, Saito M, Sakamoto K (2007). Stimulation of prostanoid IP and EP(2) receptors dilates retinal arterioles and increases retinal and choroidal blood flow in rats. Eur J Pharmacol.

[17] Uehata M, Ishizaki T, Satoh H (1997). Calcium sensitization of smooth muscle mediated by a Rho-associated protein kinase in hypertension. Nature.

[18] Narumiya S (1996). The small GTPase Rho: cellular functions and signal transduction. J Biochem.

[19] Van de Velde S, Van Bergen T, Sijnave D (2014). AMA0076, a novel, locally acting Rho kinase inhibitor, potently lowers intraocular pressure in New Zealand white rabbits with minimal hyperemia. Invest Ophthalmol Vis Sci.

